# Dietary supplementation of red-osier dogwood polyphenol extract changes the ileal microbiota structure and increases *Lactobacillus* in a pig model

**DOI:** 10.1186/s13568-021-01303-8

**Published:** 2021-10-29

**Authors:** Shugui Zheng, Jichen Song, Xia Qin, Kai Yang, Mei Liu, Chengbo Yang, Charles M. Nyachoti

**Affiliations:** 1 College of Animal Science and Veterinary Medicine, Shenyang Agricultural University, 120 Dongling Road, Shenyang, Liaoning 110866 People’s Republic of China; 2 Department of Animal Science, University of Manitoba, Winnipeg, MB R3T 2N2 Canada

**Keywords:** Red-osier dogwood extract, Polyphenols, Ileal microbiota, *Lactobacillus*, Pig

## Abstract

Red-osier dogwood (ROD) extract contains a lot of polyphenols that have the potential for modulation of gut microbiota. However, little information is available about its prebiotic properties. This study investigated the impact of ROD polyphenol extract on the ileal microbiota with dietary supplementation of ROD polyphenol extract in a pig model. The data indicated that supplementation of ROD polyphenol extract significantly increased class *Bacilli*, order *Lactobacillales* and family *lactobacillaceae*. Within family *lactobacillaceae*, *Lactobacillus* was the main responder by increasing from 5.92% to 35.09%. Further analysis showed that ROD polyphenol extract improved two species *Lactobacillus delbrueckii* and *Lactobacillus mucus*. The results of this study suggested that ROD polyphenol extract has the potential to play prebiotic role and confer health benefit through modifying gut microbiota.

## Introduction

Gut health is essential for overall physical health of humans and animals. There are an enormous number of microbes, most mainly bacteria, in human and animal intestines. Many of these bacteria, which are called commensal bacteria, are highly beneficial. Commensal bacteria play a crucial role in fermenting food, modulating the host immune response, protecting against infections, and regulation of host metabolism, etc. (Flint et al. 2012; Adam et al. 2016). Besides commensal beneficial bacteria, some harmful ones also inhabit the intestine. Actually, it is the balance between beneficial and harmful bacteria that determine the function of gut microbiota and thus health in general (Kevin et al. 2005).

Some natural substances can regulate intestinal microbial balance and promote the growth and proliferation of beneficial bacteria. By increasing the number and activity of commensal beneficial bacteria such as *Lactobacillus* and *Bifidobacterium*, these substances play an important role in promoting gut health (Ozdal et al. 2016). Red-osier dogwood (ROD, *Cornus sericea*), also called red twig dogwood, red-stem dogwood or red willow, etc., is a common native shrub mainly throughout western and northern America. It grows abundantly in the low wetlands, pastures and marginal land (Isaak et al. 2013). The plant is rich in bioactive substances such as polyphenols, which have remarkable biological activities such as antioxidant and anti-inflammatory properties. Besides, many studies have also shown that plant extracts rich in polyphenols might be used as prebiotics to regulate gut microbiota (Makkar et al. 2007; Fernando et al. 2015).

Red-osier dogwood has long been demonstrated to be effective to treat diarrhea and fever and used as native traditional medicine by the natives of North American. Feeding ROD polyphenol extract was able to reduce the incidence of diarrhea and mortality in some animal model and has been suggested to be an alternative to antibiotics used in the feed industry (Schafer et al. 2011). However, the effect of ROD polyphenol extract on the composition of gut microbiota is not well defined. Therefore, the purposes of this study were to explore the effect of dietary ROD polyphenol extract on gut microbial composition in a pig model and deepen the understanding of mechanism by which ROD polyphenol extract plays a positive effect on intestinal microbes. Moreover, in view of the anatomical, physiological and polyphagous similarities between pigs and humans, pig has been considered an ideal model to study intestinal microbiota in humans. Hence, the result of this study may help to elucidate the effect of ROD polyphenol extract on gut microbiota in humans.

## Materials and methods

### Characterization of ROD polyphenol extract

The ROD polyphenol extract was kindly donated by Red Dog Enterprise Ltd. (Winnipeg, Canada). After being defatted with hexanes and extracted as described by Isaaak et al. (2013), polyphenols content was determined by an HPLC technique according to Chen et al. (2016)

Gallic acid and methyl gallate were calibrated at a wavelength of 280 nm, but ellagic acid and quercetin were at 370 nm.

### Animals, diets and sampling

The animal experimental procedures were reviewed and approved by the University of Manitoba Animal Care Committee. All pigs were cared for according to the guidelines of the Canadian Council on Animal Care (CCAC, 2009).

A total of ten crossbred barrows (Duroc × Landrace × Large white) from Glenlea Swine Research Unit, University of Manitoba, were selected and surgically fitted with a T-cannula at the distal ileum as previously described (Wubben et al. 2001). After surgery, all the pigs were housed individually under a comfortable environment. Each pen measured 1.2 × 1.5 m was equipped with a stainless steel self-feeder and low-pressure nipple drinkers. After adaption period, the pigs with the average body weight of 88.9 ± 4.3 kg were randomly assigned to two groups including the control and treatment group with five pigs per group. A non-medicated basal diet containing 73.5% corn and 23.0% soybean meal on an air-dry basis as energy and protein sources, respectively, were formulated to meet the NRC (2012) nutrient requirements for the control group. An experimental diet for ROD polyphenol extract group was also prepared with adding 0.5% ROD polyphenol extract to replace equal amount of corn in basal diet. The ingredients and nutrients of both diets were presented in Table 1. During the 15-day experiment, the pigs were fed twice daily and individually weighed on day 0 and day 15 to calculate average daily gain (ADG). The amount of feed offered and refusals was recorded every day to determine average daily feed intake (ADFI) and then feed to gain ratio (G/F). Ileal digesta samples were collected via ileal fistula after morning feeding on day 15. Samples were divided into three subsamples and transferred to sterile sample bags. One sub-sample of the digesta was used to measure pH immediately. The second sub-sample of the digesta was kept on ice and then transferred to −20 ℃ until used for analysis of volatile fatty acids (VFA), and the third sub-sample was immediately frozen in liquid nitrogen and transferred to −80 ℃ for DNA extraction and microbial analyses.

**Table 1:** Composition and nutritional contents of control and ROD polyphenol extract supplemented diets

	Control	ROD group
Composition (%)		
Corn	73.5	73.0
Soybean meal (44%)	23.0	23.0
Vegetable oil	1.00	1.00
ROD polyphenol extract	0.00	0.50
Monocal P (Biofos)	0.50	0.50
Limestone	0.70	0.70
Iodized Salt	0.30	0.30
Vitamin-mineral premix	1.00	1.00
Chemical analysis		
DE (Kcal/kg)	3.48	3.47
ME (Kcal/kg)	3.33	3.32
NE (Kcal/kg)	2.45	2.44
Crude protein (%)	16.2	16.2
Ca (%)	0.52	0.52
Total P (%)	0.49	0.49
Av. P (%)	0.18	0.18
SID Lys (%)	0.73	0.73
SID Met (%)	0.24	0.24
SID Thr (%)	0.51	0.51
SID Trp (%)	0.16	0.16
Total polyphenols (mg/kg)	–	229.56
Gallic acid (mg/kg)	–	29.78
Methyl gallate (mg/kg)	–	182.70
Ellagic acid (mg/kg)	–	17.08
Qercetin (mg/kg)	–	ND

Values of nutrient level in the table are calculated values

ROD polyphenol extract, red-osier dogwood polyphenol extract; DE, digestible energy; ME, metabolizable energy; NE, net energy; Av. P, available phosphorus; SID, standard ileal digestibility; ND, not detected

Vitamin-mineral Premix provided per kilogram of complete diet: vitamin A 1300 IU; vitamin D_3_ 150 IU; vitamin E 11 IU; vitamin K 0.5 mg; vitamin B_1_ 1 mg; vitamin B_2_ 2 mg; vitamin B_6_ 1 mg; vitamin B_12_ 0.005 mg; pantothenic acid 7 mg; niacin30mg; folic acid 0.3 mg; biotin 0.05 mg; copper 3 mg; iron 40 mg; zinc 50 mg; manganese 2 mg; selenium 0.15 mg; iodine 0.14 mg

### Metabolites and pH analysis of ileal digesta

The concentrations VFAs including acetate, propionate, butyrate, valerate, isovalerate and total VFA in ileal digesta were analyzed by gas chromatography as previously described (Wang et al. 2011). Ileal digesta pH was measured by a pH electrode connected to a pH meter.

### Microbial DNA extraction, sequencing and data analysis

Microbial genomic DNA was extracted from each digesta sample using a commercial DNA isolation kit (Omega Biotek, Norcross, GA, USA) according to the manufacturer´s instruction. DNA was quantified using a NanoDrop (Thermo Fisher Scientific, Waltham, MA, United States). The V4–V5 hypervariable regions of bacterial 16S rRNA gene were used for sequencing. After PCR amplification, amplicons were purified using Axy Prep DNA Gel Extraction Kit (Axygen Biosciences, Union City, CA, USA). After that, the purified amplicons were pooled in equimolar and paired-end sequenced on an Illumina MiSeq platform according to the standard protocols.

The sequence data were analyzed using the QIIME (version1.9.1) software package according to the standard guideline. Paired end reads were merged by FLASH (version 1.2.11) with a maximum mismatch rate of 0.10 and the minimum overlap of 10 bp. Quality filtering was carried out and sequences were removed if their quality score was smaller than 20, their overlapped length is less than 10 bp and they contained ambiguous bases, chimeric sequences or a mismatch to primer sequences or barcode tags. The high-quality sequences were clustered into operational taxonomic units (OTUs) at a similarity of 97%. The taxonomy assignment of OTUs was determined by comparing sequences to the Green-gene. The Venn diagrams were constructed to present unique and shared fecal OTUs between control and ROD polyphenol extract groups. α-diversity indices, including Shannon index, Simpson index, Chao1, and Observed species (Obs), were calculated using Mothur version 1.39.5 (Patrick Schloss, Ann Arbor, USA). β-diversity was presented using non-metric multi-dimensional scaling (NMDS) based on Bray–Curtis distances using the GUniFrac packages. Linear discriminant analysis effect size was performed to determine the significantly differential bacteria between two groups. Gene prediction was conducted using PICRUSt 2.

The 16S rRNA sequence information in this study has been deposited into the Sequence Read Archive (SRA) database under accession no. PRJNA692786.

### Statistical analysis

All statistical analysis in this research was carried out in R 3.2.1. Welch'st-test between-group α-diversity comparisons was calculated with R. The software was also employed to calculate non-metric multi-dimensional scaling (NMDS) of (un) weighted UniFrac distances. The value of *P* < 0.05 was considered statistically significant. Spearman’s rank correlation analysis between important bacteria and metabolite profiles was performed using Graphpad Prism version 6.0 (GRAPHPAD Software, San Diego, CA, USA). Correlation was considered significant at *P* < 0.05.

## Results

### Polyphenol content of ROD polyphenol extract

The polyphenol extract contained abundant polyphenols, including 5.955 mg/g of gallic acid, 36.539 mg/g of ethyl gallate and 3.415 mg/g of ellagic acid.

### Dietary ROD polyphenol extract had no significant effect on performances in finishing pigs

There was no significant effect on the ADG, ADFI and F/G due to supplementation of ROD polyphenol extract (Table 2).

**Table 2:** Effects of ROD polyphenol extract supplementation on growth performances in finishing pigs

	Controls	ROD group	SEM	*P* value
ADG (g/d)	1051.6	1207.6	80.3	0.191
ADFI (g/d)	2813.3	2813.3	0.0	–
F/G	2.71	2.39	0.19	0.270

ADG, average daily gain; ADFI, average daily feed intake; F/G, feed to gain ratio; SEM, standard error of the mean

Data are presented as group means of five piglets per group

### The effect of dietary supplementation of ROD polyphenol extract on volatile fatty acid production and pH

To determine the effect of ROD polyphenol extract on the ileal fermentation parameters, the concentrations of acetate, propionate, butyrate, valerate, isovalerate, total VFA and ileal pH were determined. As shown in Table 3, the concentration of propionate was significantly enhanced by dietary supplementation of ROD polyphenol extract compared with the control group. However, there were no differences in acetate, butyrate, valerate, isovalerate, total VFA and ileal pH between the control and ROD polyphenol extract group.

**Table 3:** Effects of dietary ROD polyphenol extract supplementation on VFA concentration and pH of ileal digesta in finished pigs (μmol/g)

	Control	ROD group	SEM	*P* value
Acetate	94.81	65.82	19.47	0.312
Propionate	3.99	11.59	2.65	0.045
Butyrate	12.91	9.30	3.91	0.567
Valerate	0.40	1.03	0.43	0.405
Isovalerate	1.63	1.51	0.48	0.863
Total VFA	113.76	89.47	24.29	0.486
pH	6.78	6.66	0.13	0.582

VFA, volatile fatty acid

SEM, standard error of the mean

### Dietary ROD polyphenol extract increased the diversity and numbers of bacteria in ileal digesta

**Fig. 1:**
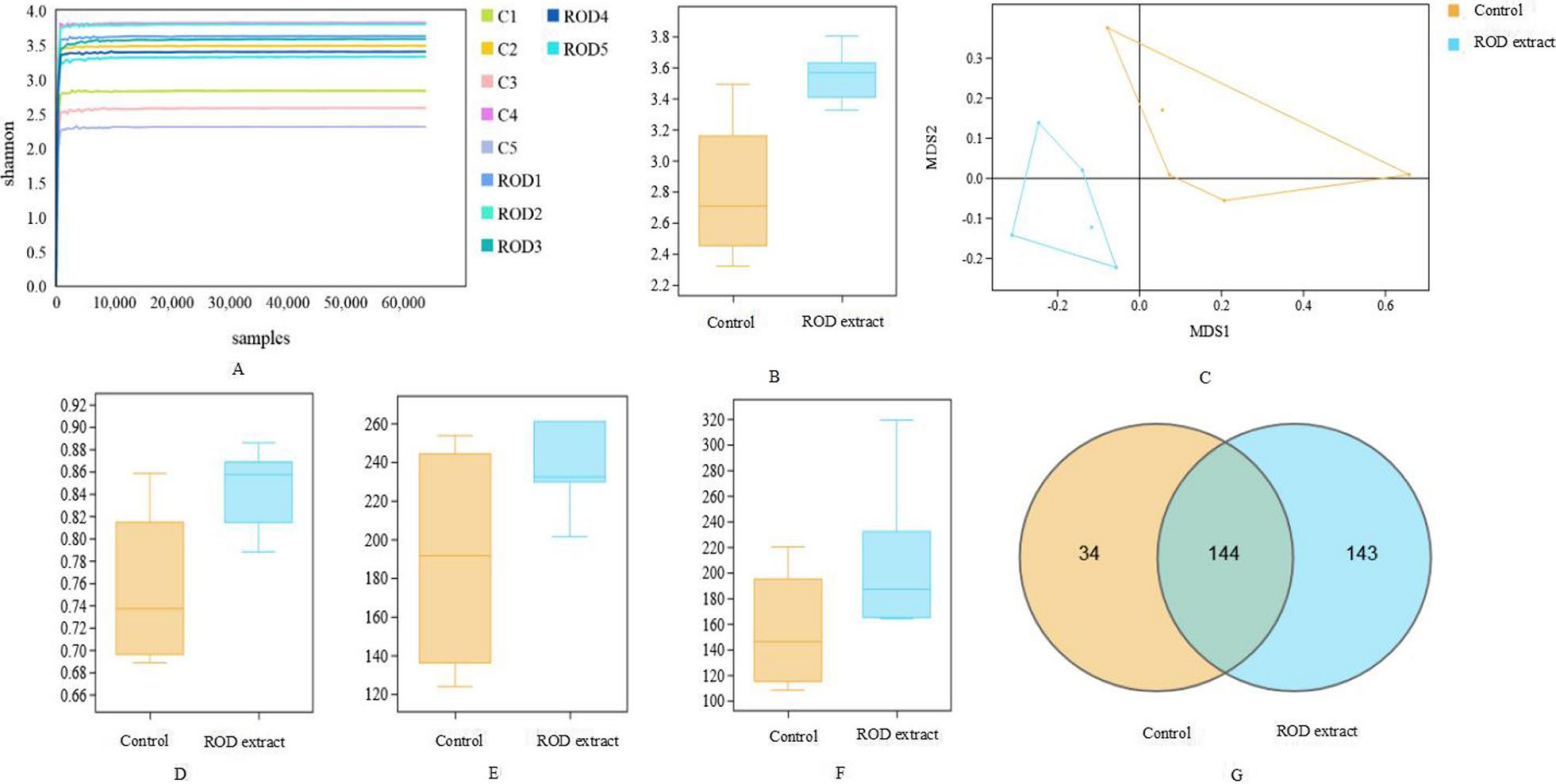
The α- and β-diversity of ileal bacterial communities in finishing pigs in the both control and ROD polyphenol extract group. Shannon–Wiener curves of ileal microbiota of finishing pigs on day 15 were shown (**A**). Bacterial diversity between the control and ROD polyphenol extract groups was assessed by the Shannon index (**B**) and Simpson index (**D**). The NMDS comparative analyses of ileal bacterial community structure between the two groups on days 15 (**C**). Bacterial richness in the control and ROD polyphenol extract groups was estimated by the Chao1 (**E**) and Robs (**F**). Shared and unique ileal OTUs between control and ROD polyphenol extract group was indicated on Venn diagrams (**G**)

Sequencing of 16S rRNA indicated that a total of 578,689 tags were obtained from the ileal samples. The stable plateau of the Shannnon-Wiener curves observed in all the samples indicated that the sequencing data were large enough to provide ample microbial information (Fig. 1A). Shannon index has indicated that dietary supplementation of ROD polyphenol extract significantly increased the α-diversity of the bacterial composition (Fig. 1B). Moreover, a statistically significant difference of bacterial composition between control and ROD polyphenol extract treatments was observed in non-metric multidimensional scaling (NMDS) analysis, a kind of β-diversity analysis, which was conducted to explore the structural variations of the gut microbiota across the samples using Bray–Curtis distance metrics (Fig. 1C).

The ileal microbiota analysis indicated a total of 321 OUTs were detected at the 97% identity. Approximately 44% OTUs was shared between the control and ROD polyphenol extract groups. Venn diagrams indicated that dietary supplementation of ROD polyphenol extract greatly increased the number of unique OTUs (Fig. 1G). This result suggested that the proportions of particular bacteria were enhanced by ROD polyphenol extract treatment.

### Dietary ROD polyphenol extract changed ileal digesta microbiota structure

**Fig. 2:**
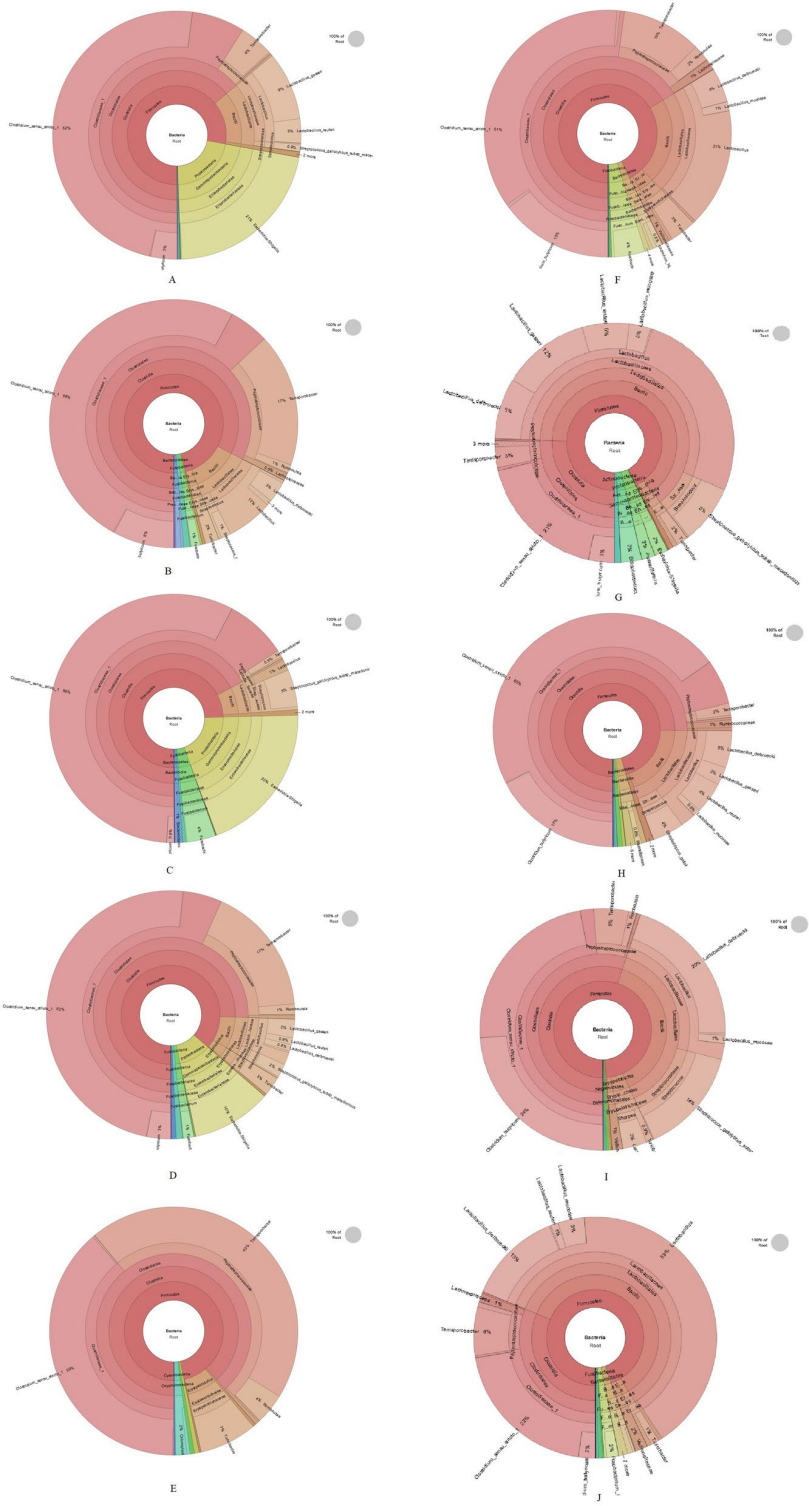
Krona pie charts of ileal microbial compositions for the five sample in control group (**A**–**E**) and the five samples in ROD polyphenol extract group (**F**–**J**). The diagram shows different taxonomic levels of ileal microbial compositions including domain, phylum, class, order, family, genus and species from the center to the outside. The fan-shaped area corresponds to the relative abundance of corresponding microbes

To further elucidate the effect of dietary supplementation of ROD polyphenol extract on pigs, microbiota structure analysis was performed. The result was showed in Fig. 2. *Firmicutes* and *Proteobacteria* were the two top phyla presented in the ileal digesta and accounted for more than 95% of all phyla in both control and ROD polyphenol extract group. At the class level, the main bacteria were *Clostridia*, *Bacilli*, and *Gammaproteobacteria* in control group. But a notable alteration in composition of microbiota was observed due to ROD supplementation in that* Bacilli* increased dramatically and replaced considerable proportions of *Clostridia* and *Gammaproteobacteria.* At the order level, *Lactobacillales* replace *Colstridiales* and *Enterobacteriales* and became significantly higher bacteria in ROD polyphenol extract group compared with the control group in which *Colstridiales* and *Enterobacteriales* accounted for greater than 85% of all orders. Further analysis at family level found that ROD polyphenol extract promoted a striking increase of *Lactobacillaceae* and replace a considerable proportion of *Clostridiaceae_1*, *Peptostreptococcaceae*, and *Enterobacteriaceae*. At genus level, dietary supplementation of ROD polyphenol extract increased *Lactobacillus* instead of some of *Clostridium_sensu_stricto_1, Terrisporobacter, Escherichia-Shigellais* and became prominent bacteria in ROD group compared with the control group.

### Dietary ROD polyphenol extract increased the abundance of beneficial bacteria

Relative abundance analysis showed that order *Lactobacillales* exclusively occupied class *Bacilli *and family *Lactobacillaceae* contained only genus *Lactobacillus.* Compared with control group, the relative abundance of *Bacilli* in ROD polyphenol extract group was enhanced by over 5 times from 7.89% to 40.60%. In the same way, the relative abundance of *Lactobacillales* in ROD treatment group was also increased by the same number. At family and genus levels, dietary supplementation of ROD polyphenol extract significantly increased the relative abundance of *lactobacillaceae* by 5.9 times from 5.92% to 35.09% compared with the control group. Similarly, the relative abundance of *Lactobacillus* in ROD polyphenol extract treatment group was more than those in the control group with the same amount (Table 4).

**Table 4:** Relative abundances of main different bacteria in ileal digesta at different taxonomic levels between control and ROD treatment group (%)

Classification levels	Taxonomic names	Control	ROD polyphenol extract	SEM	*P*-value
Phylum	*Firmicutes*	86.38	94.86	3.98	0.243
Class	*Bacilli*	7.89	40.60	7.23	0.022
Order	*Lactobacillales*	7.89	40.60	7.23	0.022
Family	*lactobacillaceae*	5.92	35.09	7.26	0.033
Genus	*Lactobacillus*	5.92	35.09	7.26	0.033
Species	*Lactobacillus delbrueckii*	0.85	10.15	2.22	0.032
	*Lactobacillus mucosae*	0.18	1.94	0.41	0.029

Supplementation of ROD treatment significantly improved *Lactobacillus delbrueckii from* 0.85% to 10.15% by nearly 12 times and notably increased *Lactobacillus mucosae* from 0.18% to 1.94% by over 10 times, respectively (Table 4).

**Fig. 3:**
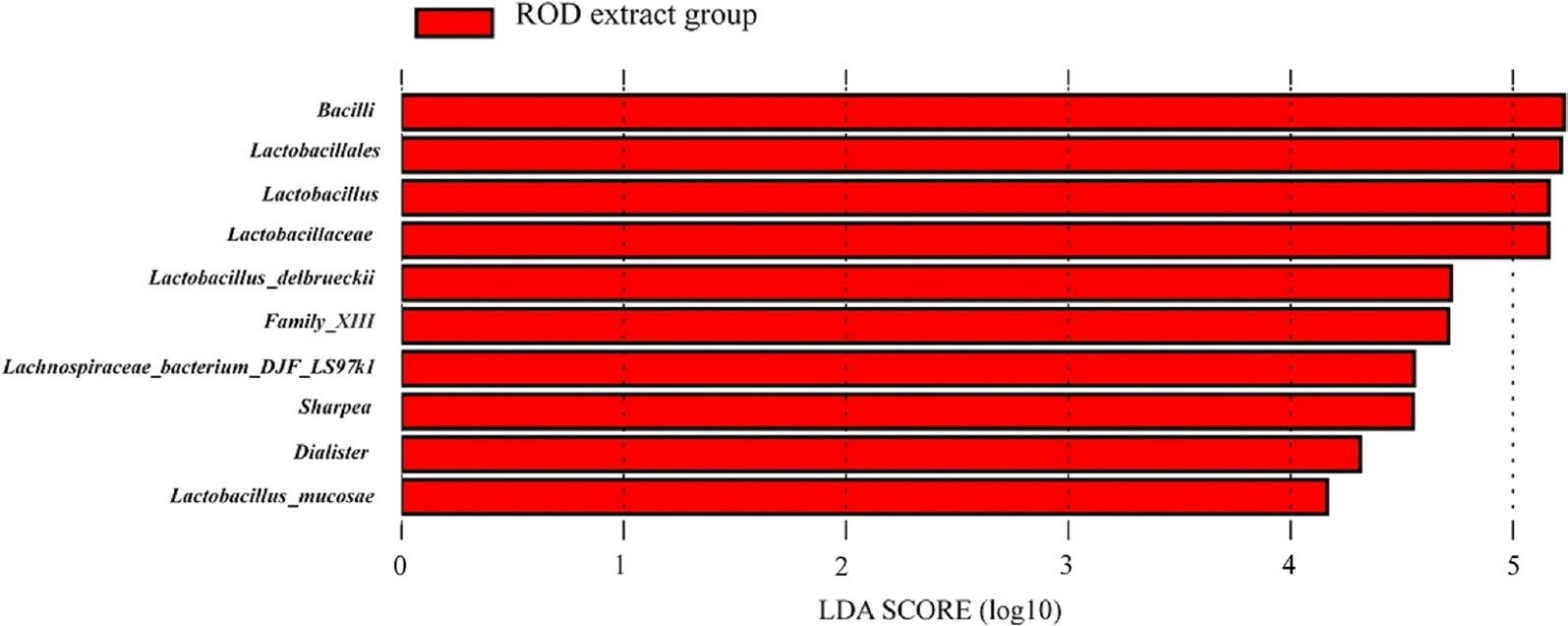
Differential enrichment of bacteria due to supplementation of ROD polyphenol extract. There is not differential enrichment of bacteria in the control group, and differential enrichment of bacteria in ROD polyphenol extract group is presented with the logarithmic LDA score of 3.0 as the cutoff value

LEfSe analysis was further carried out and discovered the dietary supplementation of ROD polyphenol extract specifically increased the abundance of some beneficial bacteria. The results showed that there was no differential enrichment of bacteria existing in the control group (Fig. 3). However, in the ROD polyphenol extract group, *Bacilli*, *Lactobacillales*, *Lactobacillaceae*, *Lactobacillus* were the dominant class, order, family and genus, respectively. Two dominant species *Lactobacillus_delbrueckii* and *Lactobacillus_mucosae* were also noted. Besides, dominant bacteria also included *Family_XIII* and two genera *sharpea* and *Dialister* as well as a species *Lachnospiraceae_ bacterium_ DJF_ LS97k1* (Fig. 3).

### Dietary ROD polyphenol extract altered metagenomic metabolic functions of ileal bacteria

**Fig. 4:**
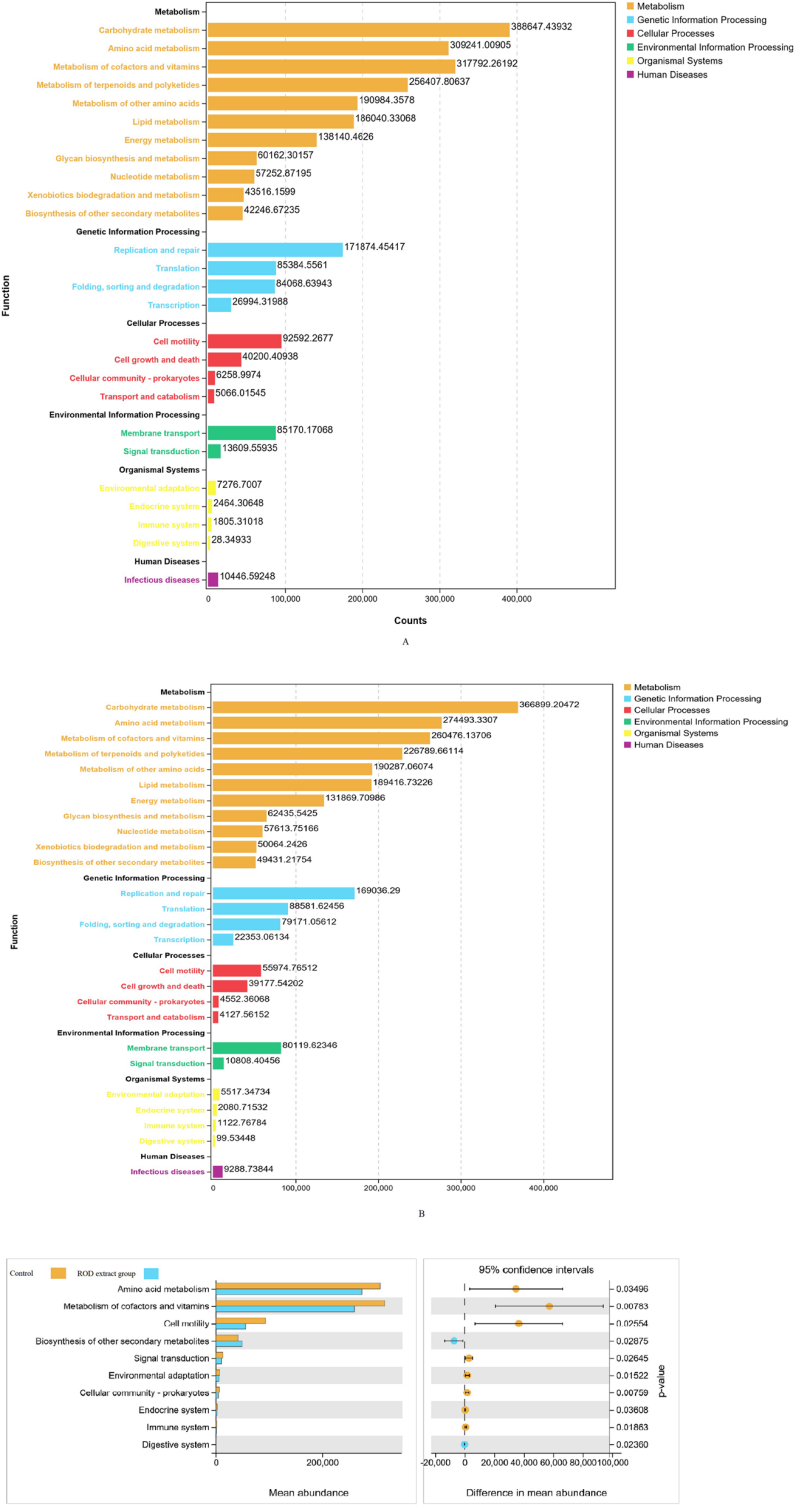
Functional diversity of ileal bacterial community of control (**A**) and ROD polyphenol extract group (**B**). Function predictions were performed by using PICRUSt 2. Functional difference between control and ROD polyphenol extract group, and only the abundances of KEGG pathways of ileal microbiota that were significantly influenced by ROD polyphenol extract are shown (**C**)

For further understand the effect of ROD polyphenol extract on intestinal bacteria, function predictions were performed using PICRUSt 2. The result shown that a total of 26 gene families were predicted to appear in all samples of both control (Fig. 4A) and ROD polyphenol extract group (Fig. 4B). Metabolomic analysis has indicated that bacteria from ileal digesta includes those involved in carbohydrate and amino acid metabolism, metabolism of cofactors and vitamins, gene replication and repair, cell motility, and membrane transport. Functional difference analysis showed that the ROD polyphenol extract group had a greater relative abundance of gene families involved in biosynthesis of other secondary metabolites (*P* < 0.05). At the same time, the ROD group had a lower relative abundance of gene families relevant to amino acid metabolism, metabolism of cofactor and vitamins, and cell motility (*P* < 0.05) (Fig. 4C).

### Correlation analysis between gut microbes and different volatile fatty acids, and pH

**Fig. 5:**
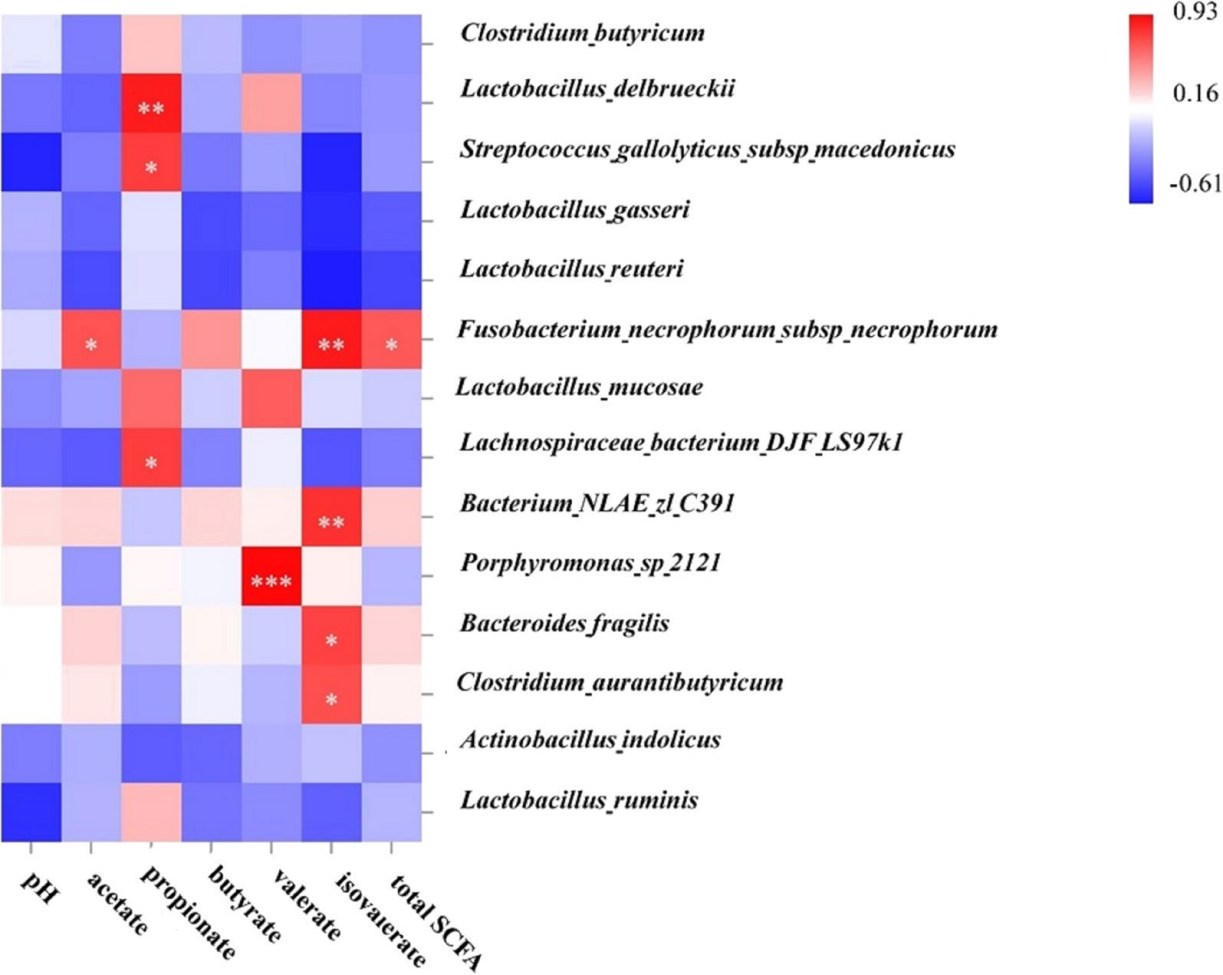
Pearson correlation analysis between bacteria and the concentration of volatile fatty acids (VFA), pH in ileal digesta of finishing pigs. The cells are colored based on the Pearson’s correlation coefficient. Red represents positive correlation. Blue represents negative correlation, and white represents no significant correlation. An asterisk represents significant correlation (*P* < 0.05), and double asterisk represents very significant correlation (*P* < 0.01)

To further elucidate whether the intestinal bacterial community has the relation with metabolites in ileal digesta, Pearson correlation analysis was carried out among different VFA, pH and the bacterial abundance at species level. As showed in Fig. 5, Propionate was positively correlated with *Lactobacillus delbrueckii, Streptococcus gallolyticus subsp macedonicus* and *Lachnospiraceae bacterium DJF LS97k1*, respectively. Valerate had obviously positive correlation with *Lactobacillus mucosae* and *Porphyromonas sp 2121*, respectively. However, isovalerate presented notably positive correlation with *Fusobacterium necrophorum subsp necrophorum*, *bacterium NLAE-zl-C391*, *Bacteroides fragilis* and *Clostridium aurantibutyricum*, respectively.

## Discussion

As the need to explore the potential of prebiotics in promoting gut health, increasing attention has been paid to the potential prebiotic effects of plant extracts other than oligosaccharides (Liu et al. 2014; Moorthy et al. 2020). The International Scientific Association for Probiotics and Prebiotics (ISAPP) proposed prebiotic as a substrate selectively utilized by host microorganisms conferring a health benefit (Swanson et al. 2020). This new definition extends the concept of prebiotic to phytochemicals, phenolics, and polyunsaturated fatty acids (Brochot et al. 2019). Studies have demonstrated that there was complicated interaction between phenolics and gut microbiota. On one hand, intestinal microbes could metabolize phenolics. Some bacterial species, such as *Lactobacillus sp., Bifidobacterium sp. Bacteroides sp. Eubacterium sp., Escherichia coli*, etc. could catalyze the phenolics metabolism (Kutschera et al. 2011). Further investigation suggested that by converting into more active biologically phenolic metabolites, Gut microbita´s metabolism could enhance the biological activity of phenolics. On the other hand, a number of evidences from in vivo and in vitro studies showed that phenolic metabolites were able to act as prebiotics and modulate the ecology of gut microbiota via promoting the proliferation of beneficial bacteria and inhibiting the growth of pathogenic microbes, consequently influencing the host health (Ozdal et al. 2016).

The complex interaction between phenolics and gut microbiota contributes greatly to the overall health of animals and humans (Gowd et al. 2019). An in vitro research showed that phenolics from green tea, black tea and oolong tea significantly influences gut microbiota by increasing the growth of *Lactobacillus* spp., *Bifidobacterium* spp. and *Enterococcus* spp while inhibiting the growth of *Prevotella, Bacteroides*, and *Clostridium histolyticum* groups (Sun et al. 2018).Several experimental evidences also showed that phenolics-rich compounds exerted the antioxidant and anti-inflammatory effects via the interaction with gut microbiota (Pandey et al. 2009; Gowd et al. 2019). In the present study, dietary supplementation of ROD polyphenol extract increased microbial α-diversity and the numbers of commensal bacteria. Clustering pattern also showed that ileal digesta samples from ROD supplemented pigs clustered away from the control. These results suggested that ROD polyphenol extract promoted more diverse and complex microbiome in the ileum. A more diverse gut microbiota may help to improve gut health and, in turn, reduce the risk of disease (Flint et al. 2012).

Moreover, dietary supplementation of ROD polyphenol extract changed the ileal microbiota structure and increased beneficial bacteria. In the present research, *Firmicutes* and *Proteobacteria* were observed to be the top two phyla in both control and ROD polyphenol extract group. This result was consistent with a previous study, where *Firmicutes* and *Proteobacteria* were two most dominant phyla in ileal digesta of pigs (Torres-Pitarch et al. 2020). At the class level, the dominant bacteria were *Clostridia*, *Gammaproteobacteria* and *Bacilli* in control group, whereas dietary supplementation of ROD polyphenol extract significantly increased the relative abundance of *Bacilli* by replacing substantial proportions of *Clostridia* and *Gammaproteobacteria*. *Lactobacillales* was the only order of class *Bacilli* observed in pigs´ ileal digesta in this study. Dietary supplementation of ROD polyphenol extract significantly increased the relative abundance of *Lactobacillales* and replaced large proportions of the *colstridiales* and *Enterobacteriales,* the two orders that accounted for the most of the orders in control group. The order *Lactobacillales* consists of six families including *Lactobacillaceae*, *Streptococcaceae*, *Leuconostocaceae*, *Enterococcaceae, Aerococcaceae*, and *Carnobacteriaceae* in taxonomy*.* Among these families, only *Lactobacillaceae* and *Streptococcaceae* were found in pigs´ileal digesta in the present study. After dietary supplementation of ROD polyphenol extract for 15 days, the relative abundance of *Lactobacillaceae* was observed to increase significantly by replacing considerable proportions of *Clostridiaceae 1*, *Peptostreptococcaceae* and *Enterobacteriaceae* in the control group. Family *Lactobacillaceae* contain only one genus *Lactobacillus* which is the major beneficial commensal inhabitant in human and animal intestines. *Lactobacillus* maintains intestinal health by production of antimicrobial substances, preventing gut colonization of enteric pathogenic bacteria (Lee et al. 2012), stimulating the mucosal immune response (Erickson et al. 2000; Hou et al. 2015) and regulating the cytokine expression (Smits et al. 2005; Frossard et al. 2007). The present study showed that dietary supplementation of ROD polyphenol extract significantly increased *Lactobacillus* by replacing considerable proportions of *Clostridium *sensu stricto* 1, Terrisporobacter* and *Escherichia Shigellais*, suggesting that ROD polyphenol extract could optimize the microbiota structure and increased beneficial bacteria.

From the perspective of taxonomy, the genus *Lactobacillus* includes 152 validly described species (Salvetti et al. 2012). The present study showed that compared with control group dietary supplementation of ROD polyphenol extract significantly increased the abundance of two species *Lactobacillus delbrueckii* and *Lactobacillus mucosae*. As type species of the genus *Lactobacillus*, *Lactobacillus delbrueckii* has long been demonstrated to have probiotic effect on human and animals (Yu et al. 2012; Lick et al. 2001; Sun et al.^2015^). It played an important role in maintaining gut health via alleviating intestinal tissue damage (Sun et al. 2015) and renovating mucosal barrier destruction (Yu et al. 2012). It has also anti-inflammatory property (Clarissa et al. 2014; Moro-García et al. 2013) and the ability to restore intestinal microbita dysfunction (Sun et al. 2015). *Lactobacillus mucosae,* however, was first isolated from pig intestines and had mucus-binding activity. It has been shown to decrease epithelial permeability, improve epithelial barrier function and provide competitive exclusion against many of pathogenic bacteria (Watanabe et al. 2010). The results of this study indicated that dietary ROD polyphenol extract increased the abundance of *Lactobacillus delbrueckii* and *Lactobacillus mucosae* in pigs´ileal digesta, suggesting the potential of ROD polyphenol extract to modulate gut health and be the promising prebiotics for animal and food industry.

The function of intestinal microbial community is closely related to microbiota structure and usually changes with changes in its composition (Mauro et al. 2013; Cao et al. 2020). In this study, results of PICRUSTs 2 indicated in finishing pigs had a variety of functions, particularly for carbohydrate metabolism, amino acid metabolism, and metabolism of cofactors and vitamins. The systems of carbohydrate metabolism, amino acid metabolism, and cofactors and vitamins metabolism are naturally present in all living cells and is crucial for metabolism of substance and energy in living organism, thereby having great significance for microbial survival. Due to the fact that ROD polyphenol extract was capable of alter gut bacteria composition, intestinal bacterial functions may also be changed. In this study, in comparison with control group, the relative abundance of gene families associated to biosynthesis of other secondary metabolites was increased in the ROD polyphenol extract group. While the relative abundance of gene families related to amino acid metabolism, metabolism of cofactor and vitamins, cell motility was decreased after ROD polyphenol extract treatment. The abundance of genes associated with the biosynthesis of secondary metabolites was increased due to supplementation of ROD polyphenol extract, suggesting that ROD polyphenol extract may have a positive impact on secondary metabolites, which perhaps is associated with the polyphenols in ROD polyphenol extract. The decreased abundance of genes associated with amino acid metabolism indicates that the ability of metabolizing amino acid might be lowered by decreasing the number of amino-acid metabolizing bacteria due to ROD supplementation. However, due to prediction limitation and complex microbial function, further research should be conducted to validate this notion.

As the molecules produced by bacteria when they ferment dietary components primarily carbohydrate inside the intestine, volatile fatty acids (VFA), mainly acetate, propionate and butyrate, are beneficial to the host health when present in sufficient quantities (LeBlanc et al. 2017). In this study, dietary supplementation of ROD polyphenol extract was shown to significantly increase the concentration of ileal propionate. This might result from the change of ileal digesta microbiota structure. We also demonstrated that there was a strong positive correlation between *Lactobacillus delbrueckii* and propionate in this study. Propionate is one of the most important VFA and it can help improve the host gut health. A research has shown that propionate exerted beneficial effects on the intestinal epithelium by improving intestinal barrier function, inhibiting inflammation, and modulating oxidative stress (Tong et al. 2016). In addition, another research also proved that propionate inhibits *Salmonella* colonization and expansion in the intestinal tract and whereby protect against *Salmonella* infections (Jacobson et al. 2018). More than that, propionate-associated health benefits were demonstrated to extend beyond the gut epithelium. Propionate was proved to lower liver lipogenesis, hepatic and plasma cholesterol levels, and carcinogenesis in other tissue. (Nishina et al. 1990; Delzenne et al. 2002; Adam et al. 2001; Jan et al. 2002). Besides, among VFAs, propionate had particularly been considered as a satiety-inducing substance with a significant effect on feeding behavior and energy intake (Ruijschop et al. 2008).

Taken together, this study demonstrated that ROD polyphenol extract has the potential to regulate gut microbial diversity, increase the abundance of beneficial bacteria, and promote to form a more balanced gut microbiota structure. Microbial metabolites, particularly propionate, were improved by the ROD polyphenol extract, exerted further beneficial effect in pig model. Our findings show that ROD may be a promising prebiotic for further application in animal and food industry.

## Data Availability

All raw sequences were submitted to the NCBI Sequence Read Archive (https://www.ncbi.nlm.nih.gov/sra/) under BioProject PRJNA692786. The data supporting the conclusion of this article are included in this article.
